# Effects of Montmorency Tart Cherry and Blueberry Juice on Cardiometabolic Outcomes in Healthy Individuals: Protocol for a 3-Arm Placebo Randomized Controlled Trial

**DOI:** 10.3390/ijerph18189759

**Published:** 2021-09-16

**Authors:** Jonathan Sinclair, Gareth Shadwell, Stephanie Dillon, Robert Allan, Bobbie Butters, Lindsay Bottoms

**Affiliations:** 1 Research Centre for Applied Sport, Physical Activity and Performance, School of Sport & Health Sciences, Faculty of Allied Health and Wellbeing, University of Central Lancashire, Preston PR1 2HE, UK; gshadwell3@uclan.ac.uk (G.S.); sdillon@uclan.ac.uk (S.D.); rallan1@uclan.ac.uk (R.A.); bbutters2@uclan.ac.uk (B.B.); 2 Centre for Research in Psychology and Sport Sciences, School of Life and Medical Sciences, University of Hertfordshire, Hatfield AL10 9AB, UK; l.bottoms@herts.ac.uk

**Keywords:** tart cherry, blueberry, cardiovascular disease, blood pressure, metabolic health

## Abstract

Cardiometabolic disease is recognized as the predominant cause of global mortality and healthcare expenditure. Whilst pharmaceutical interventions are effective in the short term, their long-term efficacy remain equivocal and their associated side-effects are concerning. Owing to their high levels of anthocyanins, Montmorency tart cherries and blueberries have been cited as potentially important natural treatment/preventative modalities for cardiometabolic disease. This study proposed a randomized controlled trial, aims to test the effects of consumption of Montmorency tart cherry and blueberry juice on cardiometabolic outcomes compared to placebo. This 20-day, parallel, single-blind, randomized, placebo-controlled trial will recruit 45 individuals, who will be assigned to receive 60 mL per day of either Montmorency tart cherry juice, blueberry juice or a cherry/blueberry flavoured placebo. The primary study outcome is the between-group difference in systolic blood pressure from baseline to post-intervention. Secondary outcome measures will be between-group differences in anthropometric, energy expenditure and substrate oxidation (during rest and physical activity), haematological, blood pressure/resting heart rate, psychological wellbeing and sleep efficacy indices. Statistical analysis will be conducted on an intention-to-treat basis. This study has been granted ethical approval by the University of Central Lancashire, Health Research Ethics Committee (ref: HEALTH 0016) and formally registered as a trial. Dissemination of the study findings from this investigation will be through publication in a leading peer-reviewed journal.

## Strengths and Limitations of this Study

- This study will be the first randomized placebo-controlled trial to examine the effectiveness of both Montmorency tart cherry and blueberry juice on cardiometabolic outcomes.
- Primary and secondary outcomes measures are central to the treatment of cardiometabolic disease and its comorbidities.

## 1. Introduction

Cardiovascular conditions, type 2 diabetes mellitus and other associated cardiometabolic disease modalities are recognized as the predominant causes of global mortality and healthcare expenditure [1]. Cardiometabolic syndrome is characterized by a range of symptoms including hypertension, obesity, insulin resistance, atherogenic dyslipidaemia, low high-density lipoproteins, high triglycerides, high levels of adiposity, high body mass index, large waist to hip ratio and poor glucose regulation [2]. To date, different pathophysiological biomarkers have emerged within the literature, with indices of oxidative stress, nitric oxide and inflammation being cited as key mechanisms that promote the clinical manifestation of cardiometabolic disease [3,4].

Traditional treatment modalities for cardiometabolic disease habitually include angiotensin-converting enzyme inhibitors, betablockers, calcium antagonists, diuretics, and lipid-lowering drugs [5]. However, whilst administration of these medications is effective in the treatment, postponement and prevention of cardiometabolic disease, their longitudinal effects and cost-effectiveness has yet to be corroborated [6], and significant negative side-effects remain common [7]. These side effects paired with recent findings show that globally 84% of adults over the age of 57 are currently taking at least one prescription medication per day and emphasize that natural cost-effective remedies are necessary for the management of cardiometabolic disease [8].

The efficacy of improved habitual dietary practice on cardiometabolic health is unambiguous, to the extent that medical organizations recommend this as the primary approach for the prevention and management of cardiometabolic disease [9]. This therefore provides a clear rationale for the adoption of dietary interventions; and indeed, moderate and sustainable improvements in health, based around effective nutritional approaches are more noteworthy, cost-effective and safer than ‘high risk’ pharmacological drugs consumed in the short-term to treat and prevent metabolic disease [10]. Diets rich in fruits and vegetables have been shown to provide protection from cardiometabolic disease [11]. However, establishing and maintaining a dietary pattern high in fruits and vegetables over a sustained duration is difficult to accomplish [12]; therefore, dietary supplementation represents a potentially more appealing treatment and prevention modality.

Anthocyanins are abundant in many fruits and vegetables and impart the dark colours found in various fruit and vegetable groups [13]. There is growing evidence that anthocyanins may confer significant improvements to cardiometabolic health [14] and Montmorency tart cherries, blueberries, strawberries, cranberries and blackcurrants (i.e., dark fruits) [15] in particular have been shown to possess high anthocyanin contents [16], although the majority of peer-reviewed literature has focused on tart cherries. Importantly, supplementation of anthocyanin rich tart cherries has been shown to effectively combat oxidative stress [17,18] and inflammation [18,19,20] and that blackberry supplementation promotes increased fat oxidation rates [21]. Improved fat oxidation rates during rest and physical activity are linked to long-term changes in body mass and composition allied to improvements in insulin sensitivity [22]. Therefore, an increased capacity to oxidize fat at rest and during moderate physical activity, instigated via anthocyanin rich supplementation, may be advantageous for yielding improvements in body composition and insulin control. Importantly, the aforementioned anti-inflammatory, anti-oxidative and substrate trafficking effects, mediated through supplementation of anthocyanin rich fruits, conveniently target the underlying chronic low-grade inflammation, pro-oxidant and lipid attenuating status that is central to cardiometabolic pathophysiology [23].

However, the findings from parallel trials investigating the effects of anthocyanin rich fruit supplementation on cardiometabolic outcomes have yielded equivocal findings. Some studies exploring the effects of tart cherry juice supplementation have shown no effect on cardiometabolic indices of blood pressure, triglycerides, insulin tolerance or cholesterol [24,25,26,27] and some have revealed improvements in systolic blood pressure and low-density lipoprotein (LDL) cholesterol [28,29]. Studies exploring the efficacy of other anthocyanin rich supplements present a similarly equivocal picture, with some demonstrating positive effects on cardiometabolic outcomes [30,31,32,33,34] and some showing no such effects [35,36,37,38]. At the current time, there has yet to be any randomized intervention studies, comparatively examining the efficacy of different anthocyanin rich fruit supplements on cardiometabolic outcomes. With some food biochemical investigations showing that anthocyanin contents in dark fruits such as blueberries are as high or even greater than in tart cherries [16], further such investigations may be of both practical and clinical relevance.

### 1.1. Aims and Objectives

The aim of the current study was to investigate the influence of 20 days of twice daily Montmorency tart cherry or blueberry juice supplementation on cardiometabolic health indices in healthy adults compared to placebo. The primary objective of this randomized trial is to examine the influence of the tart cherry and blueberry supplements on systolic blood pressure relative to placebo. Its secondary objectives are to assess if tart cherry juice and blueberry supplementation impacts on other risk factors for cardiometabolic disease.

### 1.2. Hypotheses

In relation to the primary outcome, both Montmorency tart cherry and blueberry supplement groups will mediate reductions in systolic blood pressure compared to the placebo, but no differences will be observed between supplement groups. Furthermore, for the secondary outcomes, the Montmorency tart cherry and blueberry groups will produce improvements in cardiometabolic health parameters compared to the placebo, but there will be no differences between the two supplement groups.

## 2. Materials and Methods

Described according to the updated guidelines for reporting parallel group randomized trials [39].

### 2.1. Study Design and Setting

**Figure 1:**
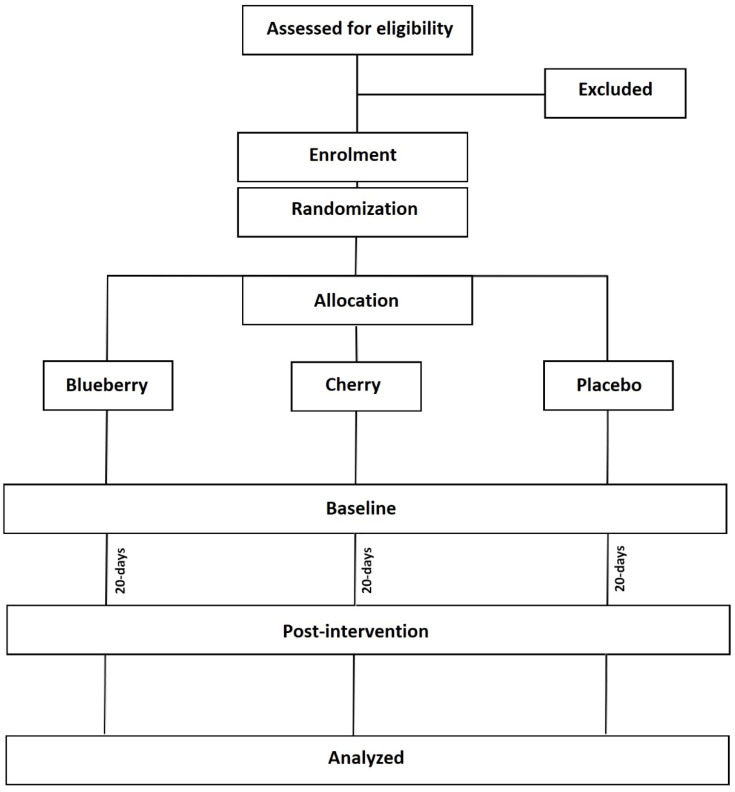
Consort diagram showing the study design.

This investigation represents a 20-day parallel, single-blind (blinded to participant) randomized placebo-controlled trial (Figure 1). After screening for eligibility and enrolment, participants will be familiarized with the testing equipment, questionnaires and procedures. Participants will then be randomized by a computer program (Random Allocation Software) to either a Montmorency tart cherry, blueberry or placebo group. Cardiometabolic health and other variables, as described in detail below, will be assessed at baseline and after 20 days (post-intervention). In agreement with previous trials of cardiometabolic health, the primary outcome measure will be the between-group difference in systolic blood pressure from baseline to post-intervention [27]. Secondary outcome measures will be between-group differences in anthropometric, energy expenditure and substrate oxidation (during rest and physical activity), haematological, blood pressure/resting heart rate, psychological wellbeing and sleep efficacy indices. All experimental visits will take place in the morning and be undertaken in a ≥10 h fasted state. Participants will also be required to arrive hydrated and to avoid strenuous exercise, alcohol, and nutritional supplements for 24 h and caffeine for 12 h prior.

### 2.2. Participants

#### 2.2.1. Inclusion Criteria

- Eighteen years of age and above
- Non-smoker
- BMI < 30
- Able to give informed consent

#### 2.2.2. Exclusion Criteria

- Pregnancy
- Sixty-five years of age and above
- Diabetes or any other metabolic/uncontrolled hypertensive conditions
- Food allergies to cherries or blueberries
- Habitual consumption of blueberries/cherries and/or blueberry/cherry products
- Not regularly taking medication or antioxidant supplements

#### 2.2.3. Sample Size

Power calculations were performed for the primary outcome variable, i.e., the between groups difference in systolic blood pressure. This showed that a total sample size of 45 will be necessary to provide 80% power to detect a minimally important clinical difference (MCID) of 6 mmHg between groups [40], with a projected standard deviation of 5.5 mmHg in each group [41], accounting for a loss to follow up rate of 10%.

### 2.3. Recruitment

It is expected that participants will predominantly be recruited from the UK city of Preston and its surrounding areas. Recruitment will be undertaken by poster promotion across the university campus, at local workplaces and also through advertisements on social media. Interested individuals will be able to contact the research team for further study information and to ask any questions associated with participation in the study. Participants will be invited to attend an eligibility, enrolment and familiarization session at the University of Central Lancashire. Written informed consent will be obtained from those willing to take part.

### 2.4. Dietary Intervention

After the conclusion of their baseline data collection session, participants will be provided with either Montmorency tart cherry, blueberry or placebo concentrate. Participants will be required to consume 30 mL of supplement diluted in 100 mL of water twice daily: once in the morning and again in the evening [27]. All supplementations will be kept refrigerated throughout. According to the manufacturer (ActiveEdge, Hanwell, UK), a 30 mL dose of Montmorency tart cherry concentrate (energy: 102 kcal, carbohydrates: 25 g of which sugars: 18 g, protein: 1.10 g and fibre: 2.6 g) is equivalent to approximately 320 mg of anthocyanins. Similarly, taking into account the manufacturers (ActiveEdge, Hanwell, UK) guidelines, a 30 mL dose of blueberry concentrate (energy: 103 kcal, carbohydrates: 22 g of which sugars: 22 g, protein: 0.2 g and fibre: 0.2 g) is equivalent to approximately 387 mg of anthocyanins. Preparation of the placebo will involve mixing 100% un-flavoured maltodextrin carbs (MyProtein, Cheshire, UK) into drinking water using a magnetic stirrer (Stuart Scientific, UK) and stir bar (Fisher Scientific, Waltham, MA, USA). Then, 666 g of maltodextrin will be added to water to create a litre of placebo concentrate, working out as 20 g of maltodextrin per 30 mL serving, closely matching the Montmorency tart cherry or blueberry concentrates. Even amounts of red and black food colouring will be added to match the colour of the Montmorency tart cherry concentrate and even amount of red, blue and black colouring to match the colour of the blueberry supplement. Either cherry or blueberry flavdrops (1 mL) (MyProtein, UK) will then added to match the required flavour. A 30 mL dose of placebo concentrate (100 kcal, carbohydrates 25 g of which sugars: 0 g, protein: 0 g and fibre 0 g) contains 0 mg of anthocyanins. This method of placebo preparation has been shown by previous intervention trials to provide an effective blinding strategy [42].

Throughout the study, the participants will be encouraged to maintain their habitual diet and exercise routines and asked to refrain from consuming any multivitamin or antioxidant supplements [24]. For their post-intervention data collection session, all participants will be asked to return any un-used supplementation to the laboratory in order to determine the actual amount of supplement/placebo that was consumed (mL), and the % compliance in each group will be reported. Furthermore, in order to examine blinding efficacy, each participant will be asked which trial arm they felt that they had been allocated to at the conclusion of their post-intervention data collection session. In both groups loss to follow up will be monitored, as will be any adverse events.

### 2.5. Data Collection

#### 2.5.1. Laboratory Visit Data

All measurements will be made at University of Central Lancashire’s physiology laboratory and will be undertaken in an identical manner on two occasions, i.e., baseline and post-intervention. The laboratories housed by the University of Central Lancashire are fully accredited by the British Association for Sport and Exercise Sciences, illustrating that they have undergone meticulous inspection and evidenced that; all instrumentation is well maintained in terms of reliability, validity and routine servicing, staff have the appropriate professional and vocational qualifications and that the requisite operational procedures for health and safety are met.

#### 2.5.2. Anthropometric Measurements

Anthropometric measures of mass (kg) and stature (m) (without shoes) will be used to calculate body mass index (kg/m^2^). Stature will be measured using a stadiometer (Seca, Hamburg, Germany) and mass will be measured using weighing scales (Seca 875, Hamburg, Germany). In addition, body composition will be examined using a phase-sensitive multifrequency bioelectrical impedance analysis device (Seca mBCA 515, Hamburg, Germany) [43], allowing percentage body fat (%) and fat mass (kg) to be quantified. Finally, waist circumference will be measured at the midway point between the inferior margin of the last rib and the iliac crest and hip circumference around the pelvis at the point of maximum protrusion of the buttocks, without compressing the soft tissues [44], allowing the waist-to-hip ratio to be quantified.

#### 2.5.3. Energy Expenditure and Substrate Oxidation

Respiratory gases will be collected throughout testing using a gas analysis system (MetaLyser 3B system, Cortex Biophysic, Leipzig, Germany). The University of Central Lancashire laboratory is air-conditioned, allowing a fixed ambient temperature of 20 °C to be maintained throughout. To quantify resting energy expenditure and substrate oxidation, participants will lay supine for a period of 20 min and data will be extracted and averaged over the final 17 min [45]. Resting fat and carbohydrate oxidation rates (g/min) will be quantified using established stoichiometric formulae (Equations (1) and (2)), assuming negligible protein utilization [46]. The amounts of each substrate expressed in g/min will be multiplied by 4 for carbohydrates and by 9 for fats [47]. These values will then be summed and subsequently multiplied by 1440 (i.e., the number of minutes per day), allowing daily energy expenditure (kcal/day) to be quantified and the percentage contribution (%) of each substrate to resting energy expenditure to be calculated.

In addition, carbohydrate and fat oxidation rates (g/min) and also the percentage contribution (%) of these substrates to energy expenditure will also be examined, during moderate intensity physical activity. Participants will walk on a treadmill (hp Cosmos Pulsar, Nussdorf, Germany) at a velocity of 4.5 km/h for a period of 6 min [47]. This walking velocity has reliably been shown to correspond to moderate exercise intensities [48]. Data will be averaged over the last minute of the 6 min test [47].

(1) $$\mathrm{Carbohydrate}\;\left(g/min\right)=4.344\times {\mathrm{VCO}}_{2}-3.061\times {\mathrm{VO}}_{2}$$

(2) $$\mathrm{Fat}\;\left(g/min\right)=1.695\times {\mathrm{VO}}_{2}-1.701\times {\mathrm{VCO}}_{2}$$

#### 2.5.4. Haematological Testing

Capillary blood samples will be collected by finger-prick using a disposable lancet after cleaning with a 70% ethanol wipe. Capillary triglyceride, total cholesterol and glucose levels (mmol/L) will immediately be obtained using three handheld analysers (MulticareIn, Multicare Medical, Washington, DC, USA) and capillary haemoglobin levels (g/L) using a single handheld analyser (HemoCue, Ängelholm, Sweden). From these outcomes, LDL cholesterol (mmol/L) will firstly be quantified using the Anandarja et al., [49] formula using total cholesterol and triglycerides as inputs. In addition, high-density lipoprotein (HDL) cholesterol (mmol/L) will also be calculated by re-arranging the Chen et al., [50] equation to make HDL the product of the formulae. Both of these approaches have been shown to have excellent similarity to their associated lipoprotein values examined using immunoassay techniques r = 0.948–0.970 [47,48]. The ratios between total and HDL cholesterol and between LDL and HDL cholesterol levels will also be determined in accordance with Millán et al. [51].

#### 2.5.5. Blood Pressure and Resting Heart Rate

Blood pressure and resting heart rate measurements will be undertaken in an upright seated position at the end of the above-described resting energy expenditure test. Both peripheral measures of systolic and diastolic blood pressure and resting heart rate will be measured via a non-invasive, automated blood pressure monitor (OMRON M2, Kyoto, Japan), adhering to the recommendations specified by the European Society of Hypertension [52]. Three readings will be undertaken, each separated by a period of 1 min [53], and the mean of the last 2 readings used for analysis.

#### 2.5.6. Questionnaires

Sleep quality is diminished in patients with cardiometabolic disease [54] and intake of dietary polyphenols [55], and supplementation of Montmorency tart cherry has been demonstrated to enhance sleep quality and symptoms of insomnolence [56,57]. Therefore, general sleep quality will be examined using the Pittsburgh sleep quality index [58], daytime sleepiness using the Epworth Sleepiness Scale [59] and symptoms of insomnolence via the Insomnia Severity Index [60]. These questionnaires will be utilized cooperatively to provide a collective representation of sleep efficacy.

Furthermore, psychological wellbeing is lower in those with cardiometabolic disease [61] and a high intake of dietary polyphenols has been shown to enhance indices of psychological wellbeing [62]. Therefore, general psychological wellbeing will be examined using the COOP WONCA questionnaire [63], depressive symptoms using the Beck Depression Inventory [64] and state/trait anxiety with the State Trait Anxiety Inventory [65]. Once again, these scales will be utilized conjunctively to provide a collective depiction of psychological wellbeing.

### 2.6. Data Management

The collection and storage of data will adhere to the standard requirements of the Data Protection Act 2018. Data will be entered onto electronic spreadsheets, which will be stored on a secure university server using Microsoft OneDrive. All data will be treated confidentially and anonymized for evaluation. Hard copies of data and documents will be kept in a locked and secure filing cabinet for the duration of the study. Following completion of the study, data will be transferred to the University of Central Lancashire Research Data Archive (CLOK), where it will be kept for 5 years. Hard copies will be disposed of confidentially and electronic data deleted after this period of time.

### 2.7. Statistical Analysis

All experimental data (with the exception of the subjective ratings of trial arm allocation) will be continuous and will therefore be presented as mean and 95% CIs. Statistical analysis of all baseline variables will be conducted to compare the three groups at baseline using linear mixed models, with groups modelled as a fixed factor and random intercepts by participants. All analyses of the intervention-based data will be performed using an intention to treat basis, and all randomized participants will be included in the final analysis as far as data collected will allow. Furthermore, in order to determine the effects of the intervention on all of the outcome measures, differences between the three groups will be examined using linear mixed models with group modelled as a fixed factor and random intercepts by participants adopted, adjusted for baseline values modelled as a continuous fixed covariate. For linear mixed models the mean difference (*b*), *t*-value and 95% confidence intervals of the difference will be presented. Finally, blinding efficacy will also be examined using a chi-squared (*Χ*^2^) test. All analyses were conducted using SPSS v27 (IBM, SPSS), and statistical significance for all analyses was accepted as the *p* ≤ 0.05 level.

## 3. Ethics and Dissemination

This study has been granted ethical approval by the University of Central Lancashire Health Research Ethics Committee (ref: HEALTH 0016) and formally registered as a trial (NCT04177238). Any required alterations to the experimental protocol will be sent for re-review/approval by the research ethics committee and amended at the trial registry. Participants who express a desire to see a summary of the trial findings will be provided with such information when the data have been analysed. Dissemination of the study findings from this investigation will be through publication in a leading peer-reviewed journal, and presentation at both national and international scientific conferences.

## 4. Conclusions and Limitations

The placebo randomized trial described in this protocol paper will explore the effects of both Montmorency tart cherry and blueberry juice on the primary and secondary outcomes pertinent to the aetiology of cardiometabolic disease and its comorbidities. As cardiometabolic conditions, they are recognized as the predominant causes of global mortality and healthcare expenditure, and the findings may provide important clinical information regarding the potential prophylactic role that anthocyanin rich fruit supplementation may play in healthy individuals.

However, like all research, the trial protocol described in this paper is not without limitations. Firstly, in order to minimize both inter and intra-subject variability, the proposed protocol will involve examining participants after an overnight fast. Therefore, it is possible that data collection will not capture the peak vasomodulatory effects of the experimental supplementation. Importantly, participants will be given instructions regarding storage/intake, and compliance to each intervention group will be quantified as any un-used supplementation will be returned and measured. However, it is ultimately not possible to control for or determine how participants actually stored or when chronologically they consumed their supplementation. Furthermore, although blood pressure will be quantified using established techniques in accordance with the European Society of Hypertension, measures will be obtained at a single time point in a laboratory environment. Therefore, 24 h continuous blood pressure monitoring may be more efficacious and representative of normal daily-living conditions and whilst also negating the potential effects of white-coat hypertension. Finally, it has been speculated that the positive effects of anthocyanin rich supplementation such as Montmorency tart cherries or blueberries on cardiometabolic health are mediated via anti-inflammatory, antioxidant and nitric oxide promoting effects. However, owing to time and cost implications, the proposed investigation will not examine pathophysiological biomarkers, meaning that the mechanistic bases for any improvements in cardiometabolic parameters will not be elucidated. Improved understanding of the mechanistic influence of anthocyanin rich supplementation on cardiovascular and metabolic health makes the expectation tenable that they can be better exploited in order to improve cardiometabolic health throughout lifespans. Therefore, future investigations beyond the study protocol described here should seek to explore and utilize the mechanistic pathways of Montmorency tart cherry and blueberry supplementation.
